# Coumarins and Their Derivatives in Animal Models of Asthma: A Systematic Review

**DOI:** 10.1111/bcpt.70210

**Published:** 2026-02-13

**Authors:** Iêda Maria dos Santos, João Lázaro de Oliveira Rocha, Edilson Beserra de Alencar Filho, Fabrício Souza Silva

**Affiliations:** ^1^ Postgraduate Program in Biotechnology State University of Feira de Santana Feira de Santana Bahia Brazil; ^2^ Laboratory of Experimental Pharmacology Federal University of the São Francisco Valley Petrolina Pernambuco Brazil; ^3^ Postgraduate Program in Biosciences Federal University of the São Francisco Valley Petrolina Pernambuco Brazil

**Keywords:** airway inflammation, asthma, coumarin, cytokine, lung

## Abstract

Asthma is a serious global health issue that affects millions of people worldwide. It is a heterogeneous disease characterized by inflammation, bronchial hyperresponsiveness and airway remodelling. Compounds from the coumarin class have been extensively documented in the literature as promising agents for asthma treatment. This review highlights the potential of coumarins and their effects in animal models of asthma. Articles from the past 10 years (2014–2024) were selected according to the guidelines of the PRISMA from the PubMed, Virtual Health Library (BVS) and ScienceDirect databases. The search strategy utilized descriptors from the Medical Subject Headings (MeSH) and Descriptors in Health Sciences (DeCS) databases. Were selected 18 articles after applying the inclusion criteria: full‐text articles published in English and Portuguese, freely accessible and investigating coumarins and their derivatives with antiasthmatic activity. The coumarins most explored by the authors were imperatorin and osthole, and the results showed that these coumarins relax airway smooth muscle, reduce inflammation and the release of Th2 cytokines IL‐4, IL‐5 and IL‐13 and increase protection against inflammation through pro‐inflammatory cells, Th1, macrophages and dendritic cells. Thus, it is concluded that coumarins and their effects, particularly imperatorin and osthole, present a promising potential in asthma management and could serve as important molecular scaffolds for future investigations.

## PLAIN LANGUAGE SUMMARY

Asthma is a chronic disease affecting millions of people worldwide, with no definitive cure, as symptoms reappear when treatment is discontinued. This study reviewed research from the past 10 years to evaluate the potential of coumarins, which are natural compounds found in plants, in the management of the disease. Among the most extensively investigated molecules, imperatorin and osthole stand out for their ability to reduce inflammation, relax airways and modulate immune responses. These findings suggest that coumarins are valuable candidates for developing novel therapies aimed at improving the quality of life of individuals with asthma.

## Graphical abstract

Mast cells drive early allergic asthma by releasing histamine, interleukins and prostaglandins, causing airway hyperresponsiveness, mucus overproduction and inflammation. These events sustain symptoms and promote structural remodelling, contributing to the progression of the disease.

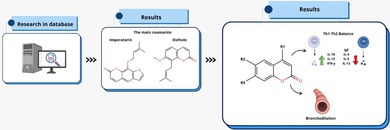

## 1 Introduction

**FIGURE 1:**
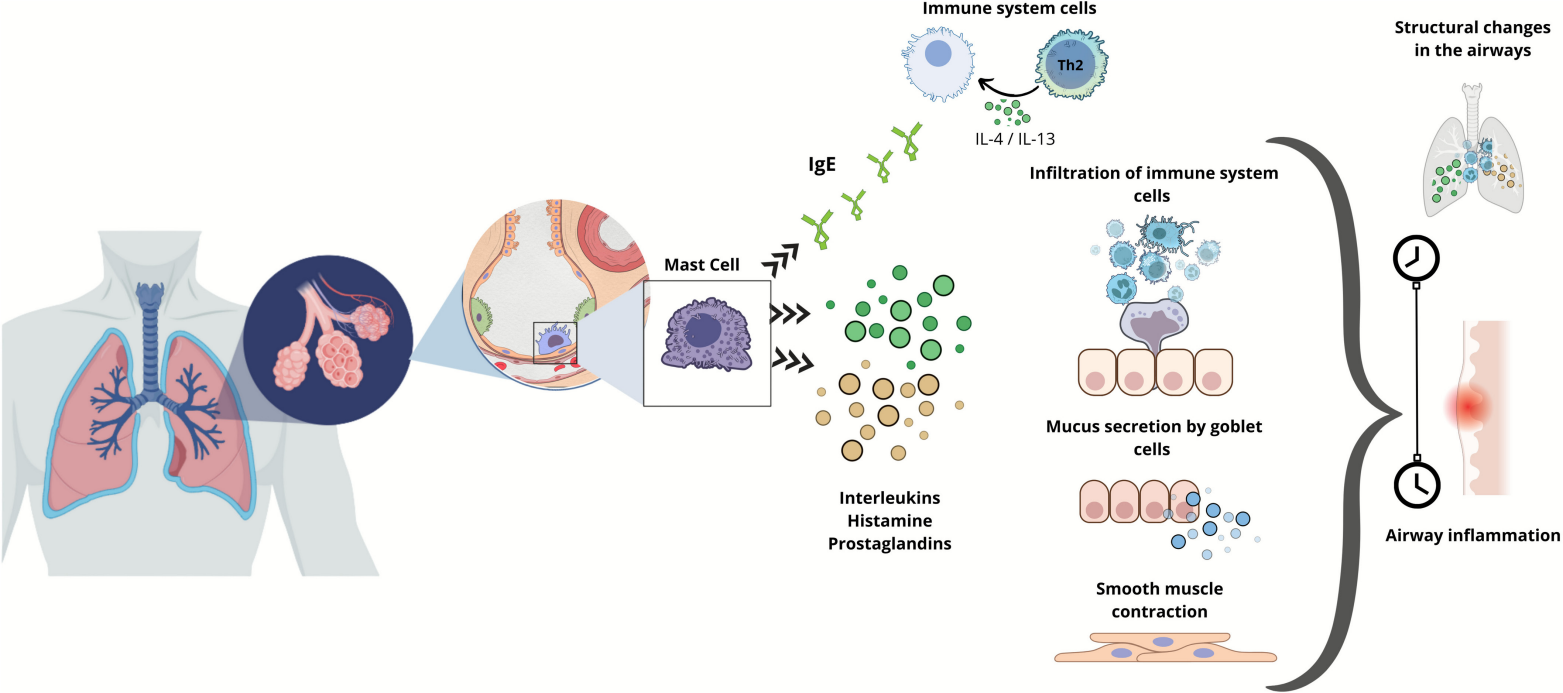
Pathophysiological features of asthma.

Asthma is characterized by chronic inflammatory processes in the airways, leading to cellular infiltration, mucus hypersecretion, oedema and smooth muscle thickening. This results in airflow obstruction, accompanied by recurrent chest tightness and breathing difficulty, causing significant suffering and mortality in patients [1]. It affects more than 300 million individuals worldwide, with increasing prevalence and incidence, leading to a significant economic burden on society. The main pathological features of asthma include airway hyperresponsiveness (AHR), inflammatory cell infiltration and excessive mucus production. Additionally, long‐term inflammation leads to structural changes in the airways (Figure 1). Epidemiological studies have shown that allergic asthma accounts for approximately 13% of all allergic diseases worldwide. It is triggered by both endogenous and environmental stimuli and can be caused by inhalation of environmental allergens, insect stings or ingestion of food allergens [2].

Mast cells (MCs) play a crucial pathophysiological role in the early stages of allergic asthma by migrating to inflamed tissues and stimulating bronchial smooth muscle contraction [3]. Inhaled allergens can bind to and activate specific receptors located on the MC membrane, triggering the release of allergic mediators such as histamine, interleukins and prostaglandins. This results in smooth muscle contraction, mucus secretion, allergic inflammation and inflammatory cell infiltration (Figure 1) [4].

Pharmacological therapy focuses on neutralising bronchoconstriction and inflammation using β_2_‐adrenergic agonists and inhaled glucocorticoids, respectively. Other therapeutic alternatives are available, including leukotriene receptor antagonists, phosphodiesterase inhibitors, muscarinic antagonists and monoclonal antibodies targeting cytokines. However, these medications may induce adverse reactions with prolonged use. Monoclonal antibodies, such as reslizumab, dupilumab and omalizumab, are indicated for the treatment of severe asthma; however, they are expensive [5, 6].

In this regard, natural products have attracted substantial attention from researchers for the development of anti‐inflammatory agents, with many bioactive compounds serving as prototypes for novel therapeutic molecules. For example, coumarins and their derivatives are an important class of natural products primarily found in the Rutaceae and Apiaceae families. These compounds are widely classified as simple coumarins, furanocoumarins, pyranocoumarins, biscoumarins and triscoumarins. The literature reports that coumarins possess a broad range of biological activities, including antimicrobial, antiviral, anticancer, anti‐inflammatory, antioxidant, anticoagulant and antiasthmatic activities [7]. In addition to these properties, coumarins are well known for their antispasmodic activity, often attributed to their ability to modulate calcium channels and smooth muscle contractility, as well as their central nervous system (CNS) effects, which include neuroprotective, anxiolytic and antidepressant actions [8].

In addition to the attractive properties mentioned earlier, coumarins possess good synthetic accessibility, offering structural diversity through molecular derivatisation and total synthesis methods. The use of natural products as a source of new drugs has been well established in the literature [8, 9, 10]. Thus, the prospecting of molecules based on the chemical structure of coumarins with potential antiasthmatic activity is of great interest, as it may represent a promising source of pharmacological alternatives for the treatment of prevalent diseases such as asthma.

## 2 Methodology

This systematic review involved a bibliographic survey of scientific data with quantitative, exploratory and descriptive methods. The research question for this study was formulated based on the guiding question: “What effect do coumarins and their derivatives have on asthma?” This review aims to compile studies on coumarins in asthma treatment from the past decade, providing robust and up‐to‐date information on their use in asthma treatment.

### 2.1 Data Selection Strategy and Search Procedures

The study design was conducted in accordance with the “Preferred Reporting Items for Systematic Reviews and Meta‐Analyses” (PRISMA) guidelines. A search was conducted for studies published in the following databases: PubMed, Virtual Health Library (BVS) and ScienceDirect. The search strategy was standardized using descriptors from the Medical Subject Headings (MeSH) and Descriptors in Health Sciences (DeCS) databases. The descriptors used for data retrieval were as follows: “cumarinas ‐ coumarin,” “asma ‐ asthma,” “pulmão ‐ lung.” Each descriptor was entered according to the database system and combined using the Boolean operators “OR” and “AND.” Only studies published between 2014 and 2024 were considered for the systematic review to present the most recent experimental data from the last decade on this topic.

### 2.2 Study Selection

The study selection process was conducted in two stages. In the first stage, titles, abstracts, and duplicate identifications were screened by two reviewers from the various databases used in this study. In case of disagreement between the reviewers, a third reviewer was consulted to make a final judgement on whether to include or exclude a study from the review. In the second stage, the reviewers assessed the inclusion of studies in the systematic review based on adherence to the eligibility criteria after reading full texts. Thus, studies were included if they met the following eligibility criteria: studies in English and Portuguese, in silico, in vitro and in vivo, addressing the effects of coumarins and their derivatives on the treatment of asthma. Duplicate, paywalled and incomplete articles were excluded, as well as studies that employed plant extracts to evaluate antiasthmatic activity.

## 3 Results and Discussion

**FIGURE 2:**
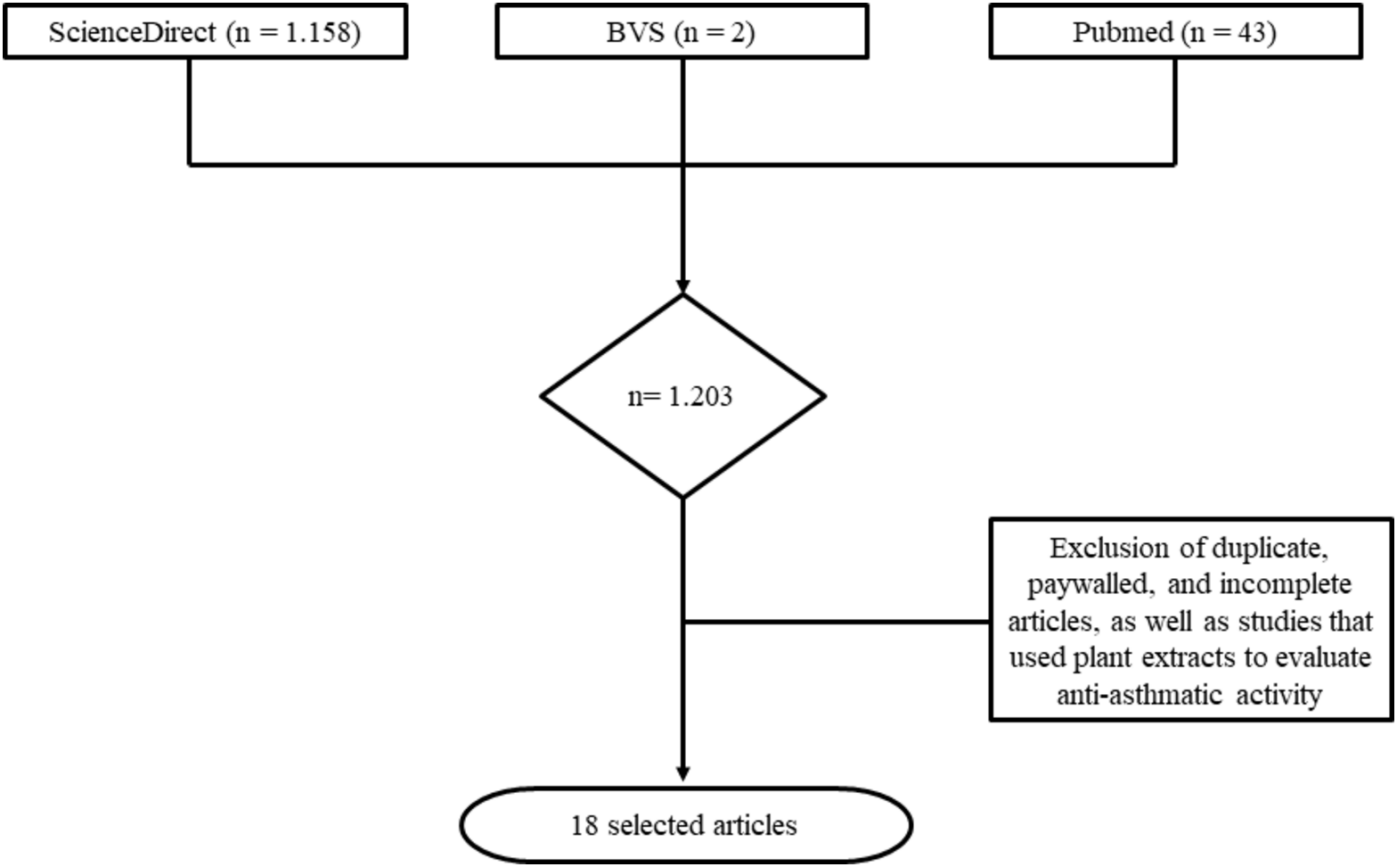
Flowchart of the studies included in this systematic review.

The search in the three defined databases resulted in 1.203 articles, with 43 identified in PubMed, 1.158 in ScienceDirect, and 2 in BVS. After applying the eligibility criteria described in the methodology, 18 articles were selected for inclusion and discussion in this systematic review (Figure 2).

**FIGURE 3:**
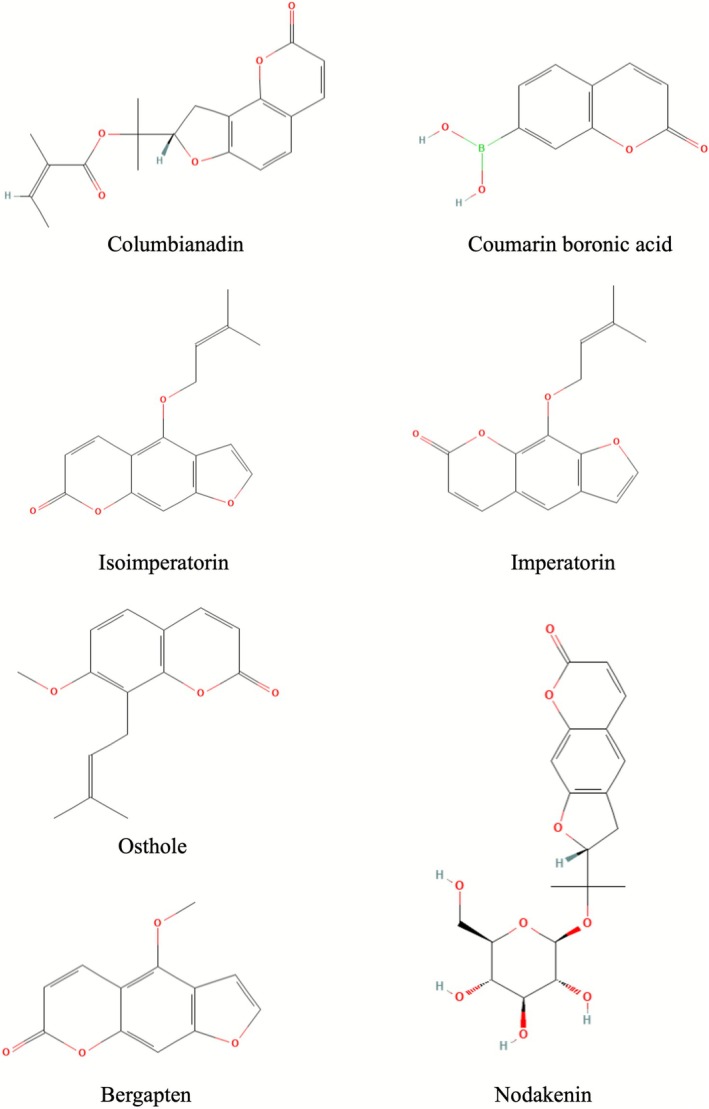
Chemical structures of coumarins from selected articles.

The data related to the general information of these studies, such as authors, year of publication, type of coumarin and/or its derivatives used in the research and a brief outcome of each article, are presented in Table 1 and Figure 3.

**TABLE 1:** General characteristics of the included studies.

Coumarin	Methodological details,	Outcome of the study	References
**Columbianadin** CAS: 5058‐13‐9 Molecular formula: C_19_H_20_O_5_ IUPAC name: 2‐[(8S)‐2‐oxo‐8,9‐dihydrofuro[2,3‐h]chromen‐8‐yl]propan‐2‐yl (Z)‐2‐methylbut‐2‐enoate	In vitro (A549, MH‐S); in vivo (mice). LPS‐induced acute lung inflammation in vivo. Control groups (without LPS); LPS; LPS + columbianadin.	In vitro inhibition of nitric oxide production in LPS‐induced A549 and MH‐S cells. In vivo reduction of pulmonary inflammation with LPS after treatment with columbianadin at doses ranging from 20 to 60 mg/kg.	[11]
**Coumarin boronic acid (CBA)** CAS: 1357078‐03‐5 Molecular formula: C_9_H_7_BO_4_ IUPAC name: (2‐oxochromen‐7‐yl)boronic acid	Clinical study in humans (analytical observation) involving patients with asthma and healthy controls. Patients with asthma (*n* = 74); healthy control (*n* = 65). Analysis performed on peripheral blood and bronchoalveolar fluids to measure oxidative stress and inflammatory markers using the real‐time detection technique of protein hydroperoxides (HP) via the coumarin boronic acid (CBA) assay.	A significant increase in reactive oxygen species in the peripheral blood of individuals with asthma was observed, specifically, the formation of protein hydroxides (HP), which occurred 35% faster and was 58% higher in asthma patients. The study highlighted how the use of CBA contributes to the management of reactive oxygen species in asthma‐related inflammation.	[12]
**Isoimperatorin** CAS: 482‐45‐1 Molecular formula: C_16_H_14_O_4_ IUPAC name: 4‐(3‐methylbut‐2‐enoxy)furo[3,2‐g]chromen‐7‐one	In vivo experimental model (BALB/c mice). Ovalbumin (OVA)‐induced allergic asthma. Control group; OVA group; OVA group + IMP (10, 30 mg/kg).	Reduction of IL‐4, IL‐5, IL‐13, eotaxin and IgE in bronchoalveolar lavage fluid (BALF), plasma and lungs. Decreases infiltration of inflammatory cells in the airways and mucus production in the respiratory tract. Reduced activity of p38 MAPK, ERK1/2 and NF‐κB, suggesting anti‐inflammatory mechanisms.	[13]
**Imperatorin (IMP)** CAS: 482‐44‐0 Molecular formula: C_16_H_14_O_4_ IUPAC name: 9‐(3‐methylbut‐2‐enoxy)furo[3,2‐g]chromen‐7‐one	In vivo, animal model (mice) with allergic asthma induced by (OVA). Groups: control (without OVA); OVA; OVA + imperatorin.	IMP attenuated Th_2_‐mediated airway inflammation by modulating dendritic cell (DC) function and directly inhibiting T cell activation.	[14]
	Allergic asthma induced by *Dermatophagoides pteronyssinus* (Der p) in mice. Groups: Negative control: mice without sensitisation/without exposure to Der p. Der p group: mice sensitized and challenged with Der p to induce asthma. IMP‐treated groups: Der p + imperatorin mice at different doses (1, 5 and 10 mg/kg).	It decreased inflammatory cell infiltration in lung tissue compared to the Der p group. It reduced endothelial cell hyperplasia and mucus hypersecretion in the airways. It decreased the total count of inflammatory cells in BALF with imperatorin (5 and 10 mg/kg). It also reduced serum IgE and IgG1, with an increase in IgG2a after treatment with imperatorin. It decreased Th2 cytokines (IL‐4, IL‐5, IL‐13) in BALF and increased Th1 cytokines (IFN‐γ, IL‐12) and IL‐10, indicating modulation of the immune response.	[15]

	MRGPRX2‐mediated mast cell activation was evaluated in murine peritoneal mast cells (MPMCs), LAD2 cells and HEK293‐Mrgprx2 cells. The IMP–MRGPRX2 interaction was analyzed by molecular docking and surface plasmon resonance (SPR), and downstream signalling by Western blot. IgE‐independent responses were examined in models of passive cutaneous anaphylaxis (PCA) and active systemic anaphylaxis (ASA), and the antiasthmatic effect of IMP in a murine model of OVA‐induced lung inflammation.	IMP reduced substance P (SP)‐induced Ca^2+^ influx and inhibited mast cell degranulation, suppressing ERK and CamKII phosphorylation and MIP‐2 and TNF‐α production. In vivo, it attenuated SP‐induced allergic asthma and OVA‐induced pulmonary inflammation by reducing mast cell activation. IMP showed high affinity for MRGPRX2 with a binding constant of 4.48 ± 0.49 × 10^−7^ M, being identified as a potential inhibitor of this receptor in allergic asthma.	[16]
The study used BALB/c mice divided into the following three groups: a control group without treatment, an OVA‐induced allergic asthma model, and an intervention group treated with imperatorin (IMP), 30 mg/kg, after asthma induction. Pathological changes in lung tissue, inflammatory cell counts in BALF, levels of inflammatory markers and oxidative stress status were evaluated. The expression of proteins from the S1PR2/STAT3 signalling pathway in the lungs was also analyzed.	Treatment with IMP: Reduced macrophage and lymphocyte infiltration in BALF; attenuated pathological damage to lung tissue; decreased levels of inflammatory and oxidative stress markers (such as MDA); Increased the activity of endogenous antioxidants (SOD and GSH‐Px). Inhibited the expression of the S1PR2/STAT3 signalling pathway, which was elevated in the asthma model. These findings indicate that IMP reversed lung damage and improved airway remodelling by suppressing S1PR2/STAT3 signalling.	[17]
**Osthole** CAS: 484‐12‐8 Molecular formula: C_15_H_16_O_3_ IUPAC name: 7‐methoxy‐8‐(3‐methylbut‐2‐enyl)chromen‐2‐one	In vitro and in vivo models (human and murine mast cells; murine models of inflammation). Groups: control without treatment (vehicle). Treatment with osthole. Different stimuli for mast cell activation: compound 48/80, substance P, LL‐37. Osthole was applied to preactivated mast cells and administered in murine models before the induction of an inflammatory response. Molecular docking studies were also used to investigate the interactions between osthole and the MRGPRX2 receptor.	Inhibition of the MRGPRX2 receptor in mast cells by osthole. Osthole affects two critical processes in MRGPRX2 receptor‐mediated mast cell activation: intracellular calcium (Ca^2+^) mobilisation and degranulation. In vivo models showed that osthole attenuates inflammation. Modelling and molecular docking data suggest that osthole acts by allosteric modulation of MRGPRX2, rather than competing directly for ligand binding sites.	[18]

	In vivo, murine model of allergic asthma (BALB/c mice). Asthma was induced by ovalbumin (OVA), with analysis of airway inflammation, hyperresponsiveness and immune response. Groups: negative control—vehicle: asthma group (OVA)—sensitisation and challenge with OVA without treatment: treatment group—OVA + osthole (5, 25 and 50 mg/kg of osthole).	Reduction of airway hyperresponsiveness in mice sensitized with OVA + osthole compared to the untreated asthmatic group. Significant decrease in Th2 cytokine levels (IL‐4, IL‐5 and IL‐13) with osthole, indicating modulation of the Th2 inflammatory response typical of allergic asthma. Increased IL‐10 and expansion of regulatory T cells (Treg) in the treated group, suggesting an anti‐inflammatory immunomodulatory effect. Altered DC maturation: osthole promoted a less pro‐inflammatory phenotype in DCs, reducing the expression of activating molecules and pro‐inflammatory cytokines. Inhibition of the proliferation of activated CD4+ T cells and suppression of Th1/Th2 responses in vitro with osthole.	[19]
	In vivo, female mice (BALB/c) with OVA‐induced asthma and in vitro, epithelial cell culture (16HBE cell line). Groups: healthy control; asthma model—(OVA) without treatment; asthma model + osthole (50 mg/kg). The viability of 16HBE cells after incubation with osthole at a series of concentrations, including 2.5, 5, 10, 20 and 40 μM for 24 h, was evaluated using the CCK‐8 cell counting kit (Beyotime, China).	TGF‐β–induced lung epithelial injury was attenuated with osthole, suggesting protection of epithelial cells. TGF‐β‐mediated epithelial‐mesenchymal transition (EMT) was inhibited by osthole, an important indication of reduced airway remodelling. Inflammation and tissue damage in asthmatic mice were alleviated with osthole treatment, indicating an anti‐inflammatory effect in vivo. In vitro, viability of 16HBE epithelial cells improved and there was a reduction in apoptosis markers with osthole compared to the group treated with TGF‐β^1^ alone.	[20]
In vivo, BALB/c mice sensitized with OVA (asthma model). Groups: control—healthy (without OVA): asthma (OVA) without osthole: asthma + osthole at two doses (25 and 50 mg/kg).	Antiasthmatic effects of osthole in mice through underlying mechanisms involving the IL‐33/ST2 pathway. The main results were a reduction in pulmonary inflammation through the infiltration of inflammatory cells, particularly eosinophils, into the lungs of mice, showing a remarkable decrease in the thickening of airway walls and in epithelial hyperplasia of the respiratory tract.	[1]

	In vivo, mice (murine model) with OVA‐induced allergic asthma. Groups: control—healthy (without OVA): OVA (induced asthma) without treatment: treatment—OVA + Osthole (25, 50 and 100 mg/kg).	Treatment with osthole reduced both inflammatory cells and Th2 cytokine levels in BLAF and serum OVA‐specific IgE. It improved pathological changes in lung tissue and suppressed NF‐κB activity in the asthma model.	[21]
	In vitro, mouse peritoneal macrophages (MPMs) were treated with osthole (20, 50 or 100 μM) for 24 h, and cell viability was measured by the MTT assay. In vivo, C57BL/6 mice with LPS‐induced acute lung injury. Groups: control—healthy: LPS—no osthole: LPS + osthole at different pretreated doses (20 and 40 mg/kg).	In vitro results indicated that the treatment inhibited the inflammatory response by blocking the nuclear translocation of NF‐κB. In vivo studies showed that osthole significantly prolonged the survival of mice with acute lung injury.	[22]
	In vivo, female mice (C57BL/6) with OVA‐induced asthma; and in vitro, alveolar macrophage cell line (NR8383) stimulated with IL‐4. Experimental groups: Healthy control: OVA asthmatic without treatment: OVA asthmatic + osthole (15 and 40 mg/kg). In vitro, treatment of NR8383 macrophages stimulated with IL‐4 with and without osthole.	Reduced macrophage activation in asthmatic lungs and CD206 expression in macrophages in the OVA + osthole treatment group compared to OVA alone. Decreased inflammatory cell infiltration and collagen deposition in the airway with osthole. Reduced pro‐inflammatory cytokines (IL‐1β, TNF‐α, MIF) in the lungs of animals treated with osthole. Suppressed the proliferation and migration of IL‐4‐induced macrophages (NR8383 cell line) with osthole in vitro studies. Decreased NF‐κB translocation (p‐IκBα and nuclear NF‐κB) in IL‐4–stimulated macrophages after osthole treatment, indicating suppression of the NF‐κB/MIF pathway.	[23]
Bronchodilator mechanisms of osthole were investigated through in silico, in vivo and in vitro experiments. In vivo, C57BL/6J female mice were exposed to 25 μg of purified house dust mite extract (HDM) and subsequently treated with osthole (25 mg/mL/10 g body weight). In vitro, mouse lung slices (PCLS) were used to evaluate the constriction‐relaxation response.	Concentration‐dependent relaxant effects were observed; the combination of osthole and albuterol resulted in greater relaxation; it reduced inflammation, and structurally, osthole binds more strongly to a specific catalytic domain of prostaglandin five (PDE4D5).	[24]
**Bergapten (BER)** CAS: 484‐20‐8 Molecular formula: C_12_H_8_O_4_ IUPAC name: 4‐methoxyfuro[3,2‐g]chromen‐7‐one	A murine model of OVA‐induced asthma was established to examine the antiasthmatic effects of BER. Calcium (Ca^2+^) influx, β‐hexosaminidase and histamine release were used to assess mast cell (MC) degranulation in vitro. RNA‐Seq was used to study gene expression profiling. RT‐PCR and Western blot were performed to examine the expression of target molecules.	It was able to suppress mast cell activation mediated by the MRGPRX2 receptor, inhibiting the expression of the nuclear protein NR4A1, thereby reducing allergic and inflammatory reactions in asthma.	[25]
**Nodakenin** CAS: 495‐31‐8 Molecular formula: C_20_H_24_O_9_ IUPAC name: (2R)‐2‐[2‐[(2S,3R,4S,5S,6R)‐3,4,5‐trihydroxy‐6‐(hydroxymethyl)oxan‐2‐yl]oxypropan‐2‐yl]‐2,3‐dihydrofuro[3,2‐g]chromen‐7‐one	In vivo, murine model of chronic allergic asthma, using BALB/c mice sensitized with OVA. Groups: Negative control: OVA‐sensitive mice receiving vehicle (without nodakenin). Treated groups: nodakenin at 3 different doses (5, 10 and 20 mg/kg) administered orally before each OVA challenge.	It reduced both the infiltration of inflammatory cells and the thickness of the bronchial wall, as well as collagen deposition in the airways of mice treated with this substance. It decreased levels of IL‐4, IL‐5, IL‐13, MMP‐2 and MMP‐9 in BALF. It also reduced specific IgE for OVA in serum.	[26]
**Coumarin derivatives**	This work reports the semisynthesis, in vitro evaluation of rat tracheal relaxation and structure–activity relationship (SAR) studies of 18 coumarins. All compounds were evaluated (0.1–500 μM) in carbachol‐induced contraction (1 μM, muscarinic cholinergic agonist) in rat tracheal rings; and theophylline (phosphodiesterase inhibitor) was used as a positive control. The evaluated compounds were used at concentrations that allowed the determination of *E*_max_ and EC50 compared to theophylline.	The results indicated that the ether derivatives 1–3, 7–9 and 13–15 exhibited the best relaxant activity (*E*_max_ = 100%), with compound 2 (42 μM) being the most potent. In the SAR, derivatives with small ether groups (methyl, ethyl and propyl) at positions 6 and 7 tend to promote greater relaxation in the trachea. Esters were less active than ethers. Additional methyl groups may modulate activity, suggesting the importance of hydrophobicity and size in the coumarin scaffold.	[27]

Lim et al. [11] reported the anti‐inflammatory effects of *Angelica decursiva* roots and its main active compound, columbianadin, a coumarin derivative, on airways. This plant has traditionally been used in medicine to treat various respiratory conditions, including cough, asthma and bronchitis. This study was conducted *both* in vitro and in vivo. In vitro, pulmonary epithelial cells (A549) and alveolar macrophages (MH‐S) were used to examine inflammatory markers such as interleukin‐6 (IL‐6) and nitric oxide (NO). In vivo, a mouse model of acute lung injury induced by lipopolysaccharide (LPS) was used, and pulmonary inflammation was measured by cell counting in bronchoalveolar lavage fluid (BALF) and histopathological analysis. Columbianadin showed strong inhibitory activity on the inflammatory response of A549 cells treated with IL‐1β and MH‐S cells treated with LPS, as well as inhibiting NO production through the negative regulation of inducible NO synthesis. Columbianadin also demonstrated significant inhibitory activity against LPS‐induced pulmonary inflammation in mice, with doses ranging from 20 to 60 mg/kg. Regarding the clinical implications of this study, the authors pointed out that columbianadin has significant anti‐inflammatory properties and suggested its potential for treating inflammatory respiratory conditions, such as asthma and chronic obstructive pulmonary disease (COPD). However, the authors stated that clinical studies are needed to confirm these findings in humans and to explore potential side effects and optimal dosing.

Bazan‐Socha et al. [12] conducted a study in humans, involving 74 patients with asthma treated at the Outpatient Clinic of the Department of Allergy and Clinical Immunology, University Hospital, Kraków, Poland. Asthma diagnosis and disease severity (mild, moderate and severe) were established based on the current guidelines of the Global Initiative for Asthma (GINA), and symptom control was assessed based on the results of the Asthma Control Test (ACT) (well‐controlled asthma, not well‐controlled asthma and very poorly controlled asthma). The authors explored the relationship between oxidative stress and asthma, focusing specifically on how an increase in reactive oxygen species (ROS) contributes to airway inflammation and remodelling in asthma. The authors employed an innovative coumarin boronic acid (CBA)‐based assay to monitor, in real time, the formation of protein hydroperoxides (HP) in the peripheral blood of asthma patients compared to healthy controls. The results showed a significant increase in systemic oxidative stress in asthmatic patients. Specifically, HP formation was 35% faster and 58% higher in patients with asthma, reflecting an increase in oxidative stress. This increase in oxidative stress was inversely correlated with lung function, suggesting that higher oxidative stress corresponds to poorer lung function. However, no significant correlations were observed between asthma severity and the histological measures of airway remodelling. Additionally, HP generation was positively associated with several inflammatory markers in both blood and bronchoalveolar lavage (BAL) fluid, including C‐reactive protein and interleukins (IL‐6 and IL‐12). Therefore, the findings suggest that proper disease control may reduce oxidative stress, thereby improving lung function and decreasing airway inflammation. In summary, this study underscores the importance of addressing oxidative stress as part of asthma management, highlighting potential therapeutic interventions that may mitigate the deleterious effects of oxidative stress in patients with asthma.

In the study conducted by Wijerathne et al. [13], the authors investigated the effects of isoimperatorin (IMP), an active furanocoumarin isolated from *Angelica dahurica* (Apiaceae), on airway inflammation and mucus hypersecretion in an ovalbumin (OVA)‐induced murine model of asthma. The authors found that IMP significantly reduced the production of interleukin IL‐4, IL‐5, IL‐13, eotaxin and immunoglobulin E (IgE) in BALF. The study further suggests that the beneficial effects of IMP may be associated with the inhibition of NF‐κB and mitogen‐activated protein kinase (MAPK) pathways. Consequently, they concluded that IMP may be a potential therapeutic agent for asthma treatment owing to its ability to attenuate airway inflammation and mucus hypersecretion, particularly in cases in which inflammation and excessive mucus production are the predominant concerns.

In the previous year, Lin et al. [14] had already studied the effects of IMP in a T helper cell type 2 (Th2)‐mediated allergic asthma model. Their results showed an increase in the population of IL‐10‐producing regulatory T cells (Tregs), an anti‐inflammatory cytokine that contributes to the suppression of the allergic inflammatory response. Furthermore, IMP altered dendritic cell (DC) function, promoting immune tolerance rather than an allergic response, which was associated with an increase in Treg cell induction. There was also a decrease in the levels of Th2 cytokines (such as IL‐4, IL‐5 and IL‐13), which are key mediators of allergic inflammation.

Hsieh et al. [15] evaluated the effect of IMP in the treatment of allergic asthma induced by *Dermatophagoides pteronyssinus* in mice. Their results showed suppression of the Th2 response, which is responsible for the production of pro‐inflammatory cytokines such as IL‐4, IL‐5 and IL‐13, leading to a decrease in the production of IgE and IgG1, which are associated with allergic responses. In contrast, Th1‐related specificity for IgG2a significantly increased in mice treated with imperatorin, which is beneficial because it not only limits inflammation but also protects against respiratory tract inflammation induced by allergic asthma. These results revealed that imperatorin balances Th1 and Th2 cytokine levels in mice with *Dermatophagoides pteronyssinus–*induced allergic asthma.

Wang et al. [16] proposed the use of IMP through a mechanism involving the inhibition of the MRGPRX2 receptor and the disruption of CamKII and ERK protein signalling, which are key components of cellular signalling cascades involved in mast cell (MC) activation. According to the authors, the G protein–coupled receptor MRGPRX2 is expressed in MCs and is one of the endogenous receptors responsible for the IgE‐independent activation of human MCs. This study combined in vitro, in vivo and computational analyses to investigate the effects of IMP on MC‐mediated allergic inflammation. The main results obtained by the authors were the inhibition of MC degranulation, and IMP reduced MC degranulation in vitro in a concentration‐dependent manner, indicating that fewer inflammatory mediators were released. Reduction in pro‐inflammatory cytokine production: IMP decreased the production of cytokines, such as IL‐4, IL‐6 and TNF‐α, which play crucial roles in the allergic inflammatory response. Suppression of cellular signalling pathways, in cellular signalling experiments, imperatorin reduced the phosphorylation of CamKII and ERK proteins, critical components of MC activation, suggesting that imperatorin directly interferes with the intracellular signalling pathway leading to inflammation. MRGPRX2 receptor blockade showed a strong affinity for the MRGPRX2 receptor, inhibiting its activation in MCs, as confirmed by in vitro experiments and molecular docking assays. In murine models of airway allergic inflammation, IMP significantly reduced lung inflammation, inflammatory cell infiltration and mucus production, demonstrating its therapeutic potential in allergic asthma.

Similar to the previous study, Callahan et al. [18] investigated the inhibition of the same MRGPRX2 receptor in MCs using another coumarin derivative, osthole. In this study, we discuss how osthole affects two critical processes in MRGPRX2 mediated MC activation: intracellular calcium (Ca^2+^) mobilisation and degranulation. Osthole significantly inhibited Ca^2+^ mobilisation after MRGPRX2 activation, directly interfering with the MC activation process, reducing MRGPRX2‐induced degranulation and preventing the inflammatory response. Osthole also reduced the production of chemokines and cytokines, such as tumour necrosis factor‐alpha (TNF‐α) and interleukin‐8 (IL‐8), which play crucial roles in inflammation. In vivo, osthole was used to assess its ability to attenuate MC‐dependent inflammation, showing a reduction in both oedema and inflammatory cell infiltration at the affected site. Molecular docking studies were conducted to investigate the interactions between osthole and MRGPRX2 receptors. This interaction was mediated by hydrogen bonds and hydrophobic interactions, which are essential for stabilizing osthole in the receptor active site. The key residues identified included amino acids located in the transmembrane region, which are responsible for intracellular signal transmission after MRGPRX2 activation. By binding to these sites, osthole blocks or reduces the ability of receptors to transmit signals that would lead to degranulation.

Wang et al. [17] also investigated the effects of IMP on airway remodelling in a bronchial asthma model, focusing on how the compound can modulate the S1PR2/STAT3 signalling pathway, which is involved in the inflammatory process and structural changes in the airways associated with asthma. The authors observed that IMP significantly reduced airway smooth muscle layer thickening and collagen deposition in an experimental asthma model. It also inhibited the activation of the S1PR2/STAT3 pathway, an important therapeutic target for treating inflammatory lung diseases, by reducing the expression levels of S1PR2 protein and phosphorylated STAT3 (p‐STAT3) in the lungs of asthmatic mice treated with the compound. This suggests that imperatorin exerts its effects by preventing the activation of this signalling pathway, which promotes inflammation and remodelling in pulmonary diseases. In addition to reducing remodelling, IMP exhibited anti‐inflammatory effects, such as decreased inflammatory cell infiltration and reduced production of proinflammatory cytokines (IL‐6, IL‐13 and TNF‐α) in the lungs. Therefore, the authors suggest that IMP is a promising therapeutic candidate, especially in specific cases where the airways become thickened due to prolonged lung injury.

Chiang et al. [19] explored the antiallergic effects of osthole in asthmatic mice and investigated its immunomodulatory actions on DCs and T cells. The main results showed a significant reduction in airway inflammation, decreased mucus production and lower eosinophil infiltration in the lungs of mice with allergic asthma. Additionally, osthole exerts important immunomodulatory effects on DC maturation and function antigen‐presenting, cells crucial for activating Th2 cells, by reducing the production of cytokines associated with this response, such as IL‐4, IL‐5 and IL‐13, which are key mediators in the pathogenesis of allergic asthma. Furthermore, this study highlighted that osthole not only suppresses the allergic response but also modulates immune system function more broadly, impacting DC maturation and function and, consequently, the adaptive immune response.

Jin et al. [22] conducted in vitro and in vivo studies to evaluate the anti‐inflammatory potential of osthole and observed a significant reduction in the production of pro‐inflammatory cytokines, such as IL‐6 and TNF‐α, following lipopolysaccharide (LPS) stimulation. Additionally, osthole inhibited both LPS‐induced degradation of the IκB‐α protein and the nuclear translocation of the NF‐κB p65 subunit, which are essential mechanisms in mediating the inflammatory response. According to the authors, the results indicate that osthole exerts a protective effect against acute lung injury, primarily through the suppression of NF‐κB–dependent inflammation, suggesting that osthole may be a promising anti‐inflammatory agent for treating septic shock and acute lung injuries.

Osthole was also studied by Tang, Liu and Zhang [20] regarding TGF‐β induced apoptosis in the pulmonary epithelium and epithelial‐mesenchymal transition (EMT) in paediatric asthma. The compound inhibited EMT, a key process in airway remodelling, by suppressing the expression of mesenchymal markers and restoring the expression of epithelial markers. In addition, osthole effectively inhibited TGF‐β–induced apoptosis in pulmonary epithelial cells. In vitro experiments showed that osthole treatment significantly reduced the expression of pro‐apoptotic proteins, such as caspases, and increased the expression of antiapoptotic proteins. Regarding signalling pathway modulation, the study indicated that osthole blocks the activation of SMAD and MAPK signalling pathways, which are triggered by TGF‐β and involved in apoptosis and EMT. This suggests that the ability of osthole to modulate these signalling pathways may open new opportunities for interventions aimed at preventing asthma progression by preserving pulmonary epithelial integrity and limiting airway remodelling.

Yang et al. [1] investigated the same compound, osthole, but focused on the IL‐33/ST2 signalling pathway, which is known to play a crucial role in inflammatory responses and asthma development. The IL‐33 receptor, ST2, is highly expressed in group 2 innate lymphoid cells (ILC2s), Th2 cells, MCs, eosinophils and natural killer (NK) cells. This study examined the antiasthmatic effects of osthole in mice to elucidate the underlying mechanisms involving the IL‐33/ST2 pathway. The main findings included a reduction in lung inflammation through decreased infiltration of inflammatory cells, particularly eosinophils, into the lungs of the treated mice. Histopathological analysis revealed a significant reduction in airway wall thickness and epithelial hyperplasia. The study also demonstrated the inhibition of IL‐33/ST2 signalling, both of which are key drivers of the inflammatory response in asthmatic mice. Additionally, there was a reduction in the levels of inflammatory cytokines associated with this pathway, including IL‐4, IL‐5 and IL‐13, which contribute to allergic inflammation and airway hyper‐responsiveness. IgE, a critical mediator of allergic responses, also showed decreased levels in osthole‐treated mice, suggesting that this compound may attenuate allergic sensitivity.

In a previous study conducted by Wang et al. [21], the authors investigated the effects of osthole on allergic asthma in a murine model of OVA‐induced asthma. In the experiment, mice were sensitized and challenged with OVA to induce asthmatic conditions characterized by airway inflammation, increased mucus production and bronchial hyperresponsiveness. Treatment with osthole resulted in a significant reduction in inflammation, mucus production and bronchial hyperresponsiveness in the treated mice compared those to in the control group. The authors also observed a decrease in the secretion of inflammatory cytokines, such as IL‐4, IL‐5 and IL‐13, suggesting that osthole may modulate the Th2 immune response, which is typically associated with allergic asthma.

Li et al. [23] demonstrated that osthole inhibited airway inflammation and macrophage activation in a murine model of OVA‐induced asthma. Osthole can suppress IL‐4 induced macrophage activation, including cell proliferation, cell migration and the production of M2 type cytokines. The inhibitory effects of this coumarin may be mediated through the NF‐κB/MIF signalling pathway (nuclear factor‐kappa B/macrophage migration inhibitory factor). The NF‐κB/MIF pathway plays a significant role in IL‐4–induced macrophage activation, where macrophage biological activities include cell proliferation, migration and M2 type cytokine secretion.

In a study by Wang et al. [24], the mechanisms by which osthole exerts bronchodilator effects were investigated to explore its potential use in improving lung function in patients with asthma. The main findings were the relaxation of small airways in lung slices from mice preconstricted with substances such as methacholine (a bronchoconstrictor agent), with the relaxant effects being dependent on the concentration of the substance. In addition to preventing muscle contraction, osthole reversed airway contraction induced by methacholine, further supporting its bronchodilatory effect. It was also shown to be effective in relaxing the airways in a manner comparable to that of conventional bronchodilators used for asthma treatment, such as theophylline and isoproterenol. The authors also investigated whether L‐type calcium channels (Cav1.2) and TRPV1 (transient receptor potential vanilloid 1) channels play a role in the airway relaxation effect induced by osthole. They used specific blockers, such as nifedipine, to test whether these channels were involved in the osthole‐induced relaxation mechanism, and the results showed that blocking these channels did not affect relaxation. This study also investigated whether osthole has a synergistic or additive effect when combined with albuterol, a β_2_‐agonist bronchodilator commonly used for asthma treatment. The combination of osthole and albuterol resulted in greater airway relaxation than albuterol alone, which could be especially useful for patients who do not respond adequately to β_2_‐agonists. In patients with asthma, prolonged use of β_2_‐agonists can lead to desensitisation of β_2_‐adrenergic receptors, making treatment less effective. The airways of mice were repeatedly treated with albuterol, leading to desensitisation of β_2_‐adrenergic receptors, which resulted in a decreased response to albuterol. Even after desensitisation, osthole was still able induced significant airway relaxation. The authors explored the molecular mechanisms by which osthole induces airway relaxation, focusing on the role of the cyclic adenosine monophosphate (cAMP) signalling pathway and protein kinase A (PKA), which is a proposed molecular mechanism underlying osthole‐induced airway relaxation. Airway smooth muscle (ASM) cells secrete PGE2, which activates EP2 and EP4 coupled to Gs to stimulate adenylate cyclase (AC) to produce cAMP, which is rapidly degraded by PDE4D (phosphodiesterase 4) in smooth muscle. Osthole inhibits PDE4D activity to amplify autocrine PGE2 signalling and increase cAMP levels, leading to ASM‐mediated airway relaxation that is dependent on cAMP/PKA. Here, osthole significantly increased intracellular cAMP levels in human airway smooth muscle cells (ASM) activate PKA, which, in addition to promoting potassium channel opening and reducing intracellular calcium concentration, phosphorylates and inhibits proteins that promote muscle contraction, thereby facilitating airway relaxation.

When the AMPc‐PKA pathway was blocked with specific inhibitors, the relaxant effect of osthole was significantly reduced, suggesting that this pathway is crucial for the mechanism of action of osthole in ASM cell relaxation. In autocrine signalling mediated by prostaglandin E2 (PGE2) through the EP2 and EP4 receptors (the main receptors involved in relaxation) in smooth muscle cells, osthole enhanced the activation of EP2 and EP4 receptors, which are coupled to the AMPc signalling pathway, promoting an increase in intracellular cAMP and, consequently, the relaxation of ASM cells. This suggests that amplifying autocrine PGE2 signalling through EP2 and EP4 receptors with osthole may help enhance airway relaxation in asthmatic conditions, where smooth muscle contractility is exacerbated by the disease. The authors found that PDE4 (phosphodiesterase 4), an enzyme that degrades cAMP, plays a central role in regulating the autocrine signalling of PGE2. When specific PDE4 inhibitors, such as rolipram, were used, they resulted in an elevation of cAMP levels and intensified the bronchodilator effect of PGE2 on ASM cells, suggesting that PDE4 acts as a negative regulator of autocrine PGE2 signalling, limiting the duration and intensity of muscle relaxation in the airways, as observed in both in vitro experiments and animal models. The authors also reported that osthole selectively inhibited the activity of PDE4D5 (phosphodiesterase D, subtype five), a specific isoform of PDE4D, with significant inhibition of this isoform compared to other PDE4 variants. This selectivity is important because the inhibition of specific PDE4 subtypes can reduce the common side effects associated with broad‐spectrum PDE4 inhibitors, such as nausea and vomiting. They also presented structural data on how osthole interacts with the PDE4D5 isoform of phosphodiesterase. Crystal structure analysis revealed that osthole binds directly to the catalytic domain of PDE4D5. This binding occurs at a specific site that is crucial for the enzymatic activity of phosphodiesterase. The crystal structure provides details about the conformation of osthole and the amino acid residues in PDE4D5 that interact with it. Regarding the inhibition mechanism, the binding of osthole to the catalytic domain prevents the enzyme from interacting with its substrate, cAMP. This inhibition results in decreased cAMP degradation, thereby increasing its intracellular levels and promoting the relaxation of airway smooth muscle cells. Structural analysis suggests that osthole may induce conformational changes in PDE4D5, which is a common feature of enzyme inhibitors. A specific region of PDE4D5, known as the upstream conserved region two (UCR2), was also examined to understand how this region influences the interaction between osthole and the enzyme. The results showed that in the presence of UCR2, osthole bound more strongly to the catalytic domain of PDE4D5, suggesting that this region may facilitate a conformation that optimizes its interaction with the compound.

Bergapten (BER) is a naturally occurring furanocoumarin isolated from plants of the Apiaceae family. Wang et al. [25] studied its inhibitory effects on MC activation mediated by MRGPRX2 in an asthma model. MRGPRX2 is a G protein–coupled receptor (GPCR) involved in MC activation that is independent of IgE and mediates drug‐induced pseudoallergic reactions. In this study, BER demonstrated the ability to suppress MRGPRX2‐mediated MC activation by inhibiting the expression of the nuclear protein NR4A1, which is essential for MC activation. The authors suggest that BER could be a novel therapeutic approach for inflammatory respiratory diseases by specifically targeting the MRGPRX2/NR4A1 signalling pathway, with the potential to reduce allergic and inflammatory reactions without significant side effects.

Xiong et al. [26] investigated the antiasthmatic potential of nodakenin, a coumarin glycoside, in an allergic asthma animal model. The authors observed decreased infiltration of inflammatory cells, particularly eosinophils, reduced levels of inflammatory cytokines such as IL‐4, IL‐5 and IL‐13 in the BALF, and a significant reduction in bronchial wall thickness and collagen deposition in the airways of mice treated with nodakenin. These results suggest that the compound may attenuate chronic structural changes in the airways and reduce inflammation and hyperresponsiveness associated with asthma progression.

Sánchez‐Recillas et al. [27] addressed the following three main aspects: semisynthesis, in vitro evaluation and structure–activity relationship (SAR) studies. The authors successfully generated a series of new coumarin derivatives that exhibited a variety of physicochemical properties, suggesting their potential biological activity in asthma treatment. The addition of ether groups (methyl, ethyl and propyl) at the seven position or at both the six and seven positions of the coumarins led to significant relaxation effects, whereas the formation of esters resulted in less active compounds. The compound that stood out the most in terms of potency demonstrated a maximum relaxant effect (*E*_max_) of 100% and an EC_50_ concentration of 42 μM, as shown in Figure 3, proving to be four times more active than theophylline (positive control). This study suggests that specific modifications to the coumarin structure can lead to compounds with enhanced bronchodilator activity, offering a promising approach for the treatment of asthma.

These findings suggest that an increase in the lipophilic chain at positions six and/or seven of the coumarin skeleton enhances the relaxant effect on rat tracheal rings. The authors proposed a pharmacophore model and designed a new molecule based on the most active molecules studied. This part of the study was based on experimental results and a description of the key chemical features shared by the most active coumarin derivatives. According to the authors, a chemical inspection of the molecular structure suggested the incorporation of a methyl group at position four, as well as ethoxy substitutions at positions six and seven.

Overall, all articles analyzed reported the effectiveness of coumarins and their derivatives in asthma treatment. However, the two compounds that stood out the most to the authors were imperatorin and osthole, with five and eight articles, respectively, although the anti‐inflammatory effects of coumarins and their derivatives are already well documented in the literature. According to the research results, both substances provided Th1‐ and Th2‐type cellular responses, as well as chemical mediators, including histamine, prostaglandins and leukotrienes, in the crucial process of asthma, which involves inflammation, followed by exacerbated smooth muscle contraction, collagen deposition and mucous secretion, potentially leading to structural remodelling when the intensity persists.

The authors also demonstrated the mechanisms through which these events occurred, highlighting how these two coumarins can act on these pathways, attenuating the main events of asthma. However, they emphasized the need for further research on the mechanisms by which coumarins and their derivatives act in the management of this disease. Regarding toxicity and/or adverse effects associated with coumarins, none of the selected articles made this clear, as they did not mention these values in their results. Although the studies included in this review did not directly address toxicological aspects, the available evidence indicates that coumarins may exhibit hepatotoxic potential in a dose‐, duration‐ and metabolism‐dependent manner. This toxicity is mainly associated with cytochrome P450–mediated metabolism and the formation of reactive metabolites, leading to oxidative stress, mitochondrial dysfunction and hepatocellular injury, particularly in animal models. In humans, coumarin‐induced hepatotoxicity appears to be rare and generally reversible, with reports of transient elevations in liver enzymes in a small proportion of individuals, highlighting the importance of monitoring liver function during prolonged or high‐dose therapy [28, 29]. In conclusion, coumarins and their derivatives are promising candidates for asthma treatment. However, the development of more studies involving these compounds is crucial, as they could provide more comprehensive information and strengthen the approach to treating asthma.

## 4 Conclusion

The bibliographic search conducted in this systematic review on the antiasthmatic effects of coumarins and their derivatives yielded a total of 18 articles involving in silico, in vitro and in vivo studies. Among these articles, special attention was given to two coumarins, imperatorin and osthole, which were explored in five and eight articles, respectively, for their airway‐relaxing, anti‐inflammatory and structural effects on the airways, which are important in asthma. The authors also showed that, as with some signalling pathways, CamKIL/ERK, S1PR2/STAT3, SMAD/MAPK, MRGPRX2/NR4A1 and AMPc/PKA, specific receptors such as Cav1.2, TRPV1 and PDE4D5 are important when activated or inhibited by coumarins to achieve effective responses in asthma. In conclusion, the promising results catalogued in this systematic review, along with the lack of data on both toxicity and adverse effects, as well as the exploration of other coumarin structures, given their well‐documented anti‐inflammatory effects in the literature, suggest that this is a research area with significant potential for exploration and expansion.

## Funding

This study was supported by Conselho Nacional de Desenvolvimento Científico e Tecnológico (grant number: 311940/2022‐6) and Fundação de Amparo à Ciência e Tecnologia do Estado de Pernambuco (grant number: APQ‐1037‐2.10/24).

## Conflicts of Interest

The authors declare no conflicts of interest.

## Data Availability

The data that support the findings of this study are available from the corresponding author upon reasonable request.
